# Early diagnosis of Lemierre syndrome using targeted next-generation sequencing combined with metagenomics capture: A case report and literature review

**DOI:** 10.1097/MD.0000000000046988

**Published:** 2026-01-09

**Authors:** Qiuyan Zhu, Qiming Liu

**Affiliations:** a Department of Respiratory and Critical Care Medicine, Suining County People’s Hospital, Suining County, Xuzhou City, Jiangsu Province, China.

**Keywords:** early diagnosis, *Fusobacterium necrophorum*, Lemierre syndrome, metagenomics capture, targeted next-generation sequencing technology

## Abstract

**Rationale:**

Lemierre syndrome (LS) is a rare but life-threatening complication of acute oropharyngeal infections. It is characterized by septic thrombophlebitis of the internal jugular vein and subsequent metastatic abscess formation. The most common causative pathogen of LS is *Fusobacterium necrophorum* (FN). This paper presents the case of a 17-year-old female patient with LS, in whom FN was rapidly detected and LS was diagnosed using targeted next-generation sequencing (tNGS) combined with metagenomics capture (MetaCAP). This approach enabled timely detection of FN and guided appropriate treatment.

**Patient concerns:**

The patient, a female 17-year-old student, experienced a fever after catching a cold, with a peak temperature of 39.8 °C on May 22, 2024, accompanied by chills and shivering, sore throat, right chest pain, back pain, cough, and hemoptysis.

**Diagnoses:**

The patient was initially diagnosed with non-severe community-acquired pneumonia at admission on May 26, 2024. She was finally diagnosed with LS after FN was detected using bronchoalveolar lavage fluid tNGS combined with serum MetaCAP.

**Interventions:**

The patient received targeted antimicrobial therapy and thorough thoracic drainage in the shortest time after being definitely diagnosed with LS using tNGS and MetaCAP technologies.

**Outcomes:**

The clinical symptoms of the patient were significantly improved. A chest computed tomography scan on July 15, 2024 indicated complete resolution of exudates and solid lesions in both lungs.

**Lessons:**

This case underscores the significant role of tNGS combined with MetaCAP in the early detection of FN and timely diagnosis of LS, systematically explores the epidemiology, clinical features, diagnosis and treatment of LS, thus providing a reference for clinicians to rapidly diagnose and treat LS.

## 1. Introduction

Lemierre syndrome (LS) is a rare but life-threatening complication of acute oropharyngeal infection. It typically originates from a pharyngeal infection that extends into the parapharyngeal space, which further causes septic thrombophlebitis of the internal jugular vein and subsequent metastatic abscess formation.^[1]^ A strong index of suspicion is required for diagnosis, as atypical manifestations often lead to misdiagnosis or missed diagnosis of LS by clinicians. The mortality rate of LS patients is about 2% to 4%, emphasizing the necessity of early identification and treatment.^[2]^ The most frequently implicated pathogen of LS is *Fusobacterium necrophorum* (FN). As a strictly anaerobic bacterium, FN has a higher missed detection rate in routine microbiological cultures, posing a significant challenge for timely and accurate diagnosis of this disease.^[3]^ This paper reports a case of LS in a 17-year-old woman, who visited the hospital due to fever, sore throat, cough, and hemoptysis. A definite diagnosis of LS was rapidly achieved after FN was detected by the combined use of bronchoalveolar lavage fluid (BALF) targeted next-generation sequencing (tNGS) and serum metagenomics capture (MetaCAP). This allowed for prompt targeted antibiotic treatment, thus resulting in a favorable patient outcome. This case suggests that tNGS and MetaCAP techniques hold significant value for the early diagnosis of LS.

## 2. Case presentation

**Figure 1.**
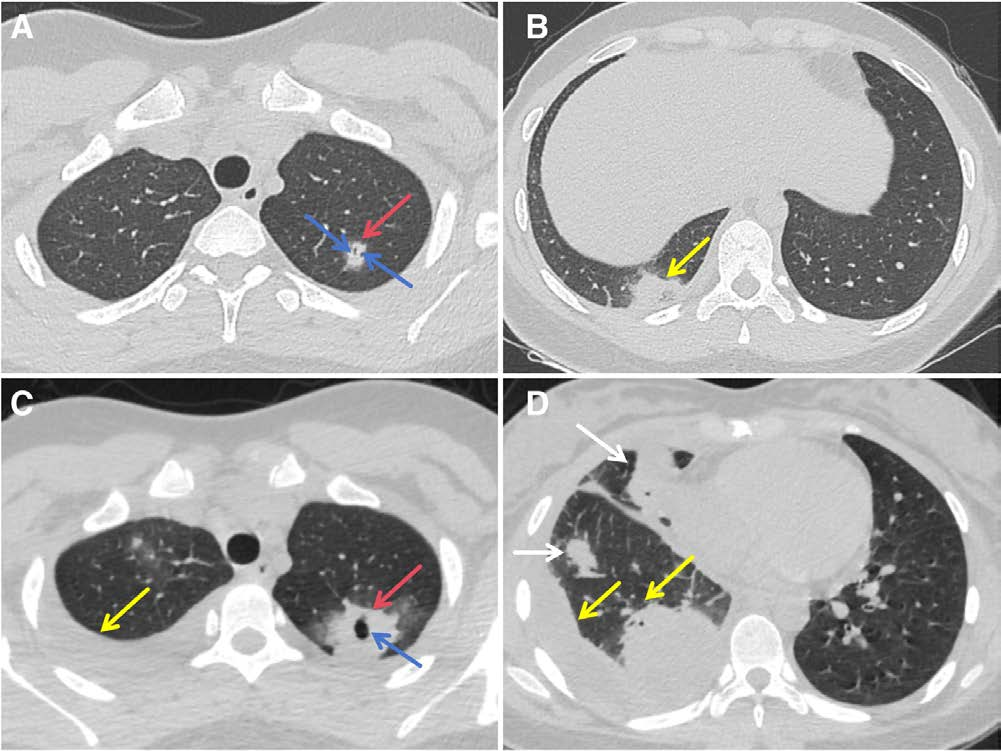
Changes in chest CT findings of the patient before a definite diagnosis of Lemierre syndrome. (A and B) The chest CT findings on May 25, 2024 revealed a nodule (A, red arrow) with 2 small cavities (A, blue arrows) in the left upper lung, and a nodule (B, yellow arrow) in the right lower lung, with no significant abnormalities in the remaining lungs. (C and D) After admission, the disease progressed rapidly, and the chest CT findings on May 29, 2024 revealed that the nodule in the left upper lung was enlarged compared with the previous one (C, red arrow) and the cavity was enlarged (C, blue arrow), and the pleural effusion appeared in the right upper lung (C, yellow arrow). Solid lesions (D, white arrows) and encapsulated pleural effusions (D, yellow arrows) occurred in the right lower lung. CT = computed tomography.

The patient was a 17-year-old female student from an urban area, without bad habits and histories of smoking, substance use, or exposure to plants or animals. The patient had a history of recurrent tonsillitis, and had visited the department of Ear, Nose, and Throat of a hospital, wherein she was advised to undergo a tonsillectomy by an otorhinolaryngologist, which she had deferred. The patient had a history of allergy to penicillin and cephalosporins. She was admitted to our hospital on May 26, 2024 due to 4 days of fever and sore throat, accompanied by 1 day of cough with hemoptysis. On May 22, 2024, the patient had fever after developing a cold, with a peak temperature of 39.8 °C, accompanied by chills and shivering. The fever pattern was irregular, accompanied by sore throat, right chest pain, back pain, and no shortness of breath. She was initially treated with azithromycin at a local hospital, with no significant improvement. On the early morning of May 25, 2024, the patient presented to the emergency department of our hospital, then she underwent a chest computed tomography (CT) scan, which indicated a nodule with 2 small cavities in the left upper lung (Fig. 1A) and a nodule in the right lower lung (Fig. 1B). No obvious abnormalities were detected in the remaining lungs. She was advised to stay in our hospital, but her family members insisted that she should be transferred back to the local hospital for continuous intravenous treatment and observation. On the afternoon of the same day, the patient developed a cough with white phlegm, accompanied by hemoptysis, which was manifested as blood in the sputum, with persistent and recurrent fever. Therefore, the patient was hospitalized in our hospital for further diagnosis and treatment on the early morning of May 26, 2024. During the course of the disease, the patient had no neck pain, neck stiffness, torticollis, dysphagia, trismus, or limited mouth opening, and she reported having a poor mental state, no night sweats, no obvious chest tightness or shortness of breath, no abdominal pain and diarrhea, no bleeding in the skin and mucous membranes, no redness and swelling of the joints, poor appetite, decreased physical strength, and normal defecation and urination.

The patient was admitted to our hospital with clear consciousness and poor spirit. Physical examination: body temperature 38.4 °C, heart rate 98 beats/min, respiratory rate 20 breaths/min, blood pressure 132/65 mm Hg, finger oxygen saturation 98% (breathing room air). She had no cyanosis in her lips and mouth, no enlargement of bilateral cervical lymph nodes or tonsils, no obvious distension of bilateral jugular veins, no hepatojugular reflux sign, and no murmurs in the neck and thyroid area. The patient’s breath sounds were slightly diminished bilaterally, with scattered moist rales audible on auscultation. Auscultation revealed no cardiac murmurs, no abdominal abnormalities, and no lower limb edema. The patient was initially diagnosed with non-severe community-acquired pneumonia.

On the day of admission on May 26, 2024, the blood and sputum samples were collected for laboratory tests. Routine blood test: leukocyte count 9.26 × 10^9^/L, neutrophil count 7.75 × 10^9^/L, neutrophil percentage 83.70%, lymphocyte percentage 10.20%, eosinophil count 0.02 × 10^9^/L, eosinophil percentage 0.20%, hemoglobin 114 g/L, hematocrit 34.90%, C-reactive protein 116.49 mg/L, high-sensitivity C-reactive protein > 10.00 mg/L, procalcitonin 1.4931 ng/mL, interleukin-6 0.5168 ng/mL, erythrocyte sedimentation rate 35 mm/h. Coagulation function test: prothrombin time 13.4 seconds, activated partial thromboplastin time 35.1 second, thrombin time 17 seconds, fibrinogen 4.92 g/L, prothrombin time activity 78.9%, D-dimer 1.01 µg/mL. Five joint tests for respiratory pathogens including *Mycoplasma pneumoniae* IgM, *Chlamydia pneumoniae* Ig, respiratory syncytial virus, adenovirus IgM, and Coxsackievirus B IgM showed negative results. Sputum culture and tuberculosis sputum smear test showed negative results. Serum Aspergillus antigen test also showed negative results.

A follow-up chest CT scan on May 29, 2024 suggested an enlarged nodule with a cavity in the left upper lung and pleural effusion in the right upper lung (Fig. 1C), and encapsulated pleural effusions and solid lesions in the right lower lung (Fig. 1D). Then, the thoracentesis was performed to drain out the pale yellow pleural effusion with a small amount of purulent fluid. Pleural effusion test: color: light red; clarity: turbid; erythrocytes: 1.6 × 10^9^/L; leukocytes: 1.65 × 10^9^/L; qualitative test of protein: positive; mononuclear cells: 25%, multinuclear cells: 75%; appearance: non-clotted. Pleural fluid biochemistry: lactate dehydrogenase: 10,462 U/L; adenosine deaminase: 199 U/L, glucose: 1.00 mmol/L; pleural fluid protein: 55.70 g/L. These findings were consistent with empyema. However, pleural fluid culture revealed negative results.

Bronchoscopy was performed on May 27, 2024, and a BALF sample was collected and sent to Nanjing Jinyu Clinical Laboratory for tNGS detection. FN (sequence number [Seq. No.] 10,461), *Aspergillus fumigatus* (Seq. No. 8093), *Candida albicans* (Seq. No. 2943), a small amount of *Haemophilus influenzae* (Seq. No. 531), and *Streptococcus anginosus* (Seq. No. 291) were detected from BALF on May 28, 2024 using tNGS, details of which were shown in Table 1. Given the patient’s adolescent age, initial symptoms such as fever, sore throat, cough, and hemoptysis, along with chest CT findings revealing multiple nodular lesions in both lungs and the detection of FN by tNGS, a diagnosis of LS was established. Bilateral cervical vascular ultrasound was performed immediately on the same day, and no internal jugular vein thrombosis was observed.

**Table 1:** Results of alveolar lavage fluid tNGS detection and serum MetaCAP detection.

Type of detection		Pathogen	Generic name		Species name			Seq. no.
Alveolar lavage fluid tNGS detection		Bacteria (G‐)	Fusobacterium		*Fusobacterium necrophorum*			10,461
			Haemophilus		*Haemophilus influenzae*			531
		Fungus	Candida		*Candida albicans*			29,431
			Aspergillus		*Aspergillus fumigatus*			8093
		Bacteria (G+)	Streptococcus			*Streptococcus pharyngitis* group		291
		Viruses, parasites and other special pathogens were not detected						
Serum detection	MetaCAP	Bacteria (G‐)		Fusobacterium			*Fusobacterium necrophorum*	58
		Cytomegalovirus					Cytomegalovirus	154
		Lymphocryptovirus					Epstein-barr virus	36
		Fungi, viruses, parasites and other special pathogens were not detected						

MetaCAP = metagenomics capture, Seq. No.= sequence number, tNGS = targeted next-generation sequencing.

A blood sample was taken from the patient on May 30, 2024 and sent to Nanjing Jinyu Clinical Laboratory for pathogen detection using MetaCAP. FN (Seq. No. 58, 93% confidence interval [CI]), cytomegalovirus (Seq. No. 154, 99% CI), and Epstein-Barr virus (Seq. No. 36, 99% CI) were detected from the serum sample using MetaCAP. The details were shown in Table 1, which further confirmed the diagnosis of LS in the patient. To our knowledge, this is the first reported case of LS diagnosed using MetaCAP from serum.

**Figure 2.**
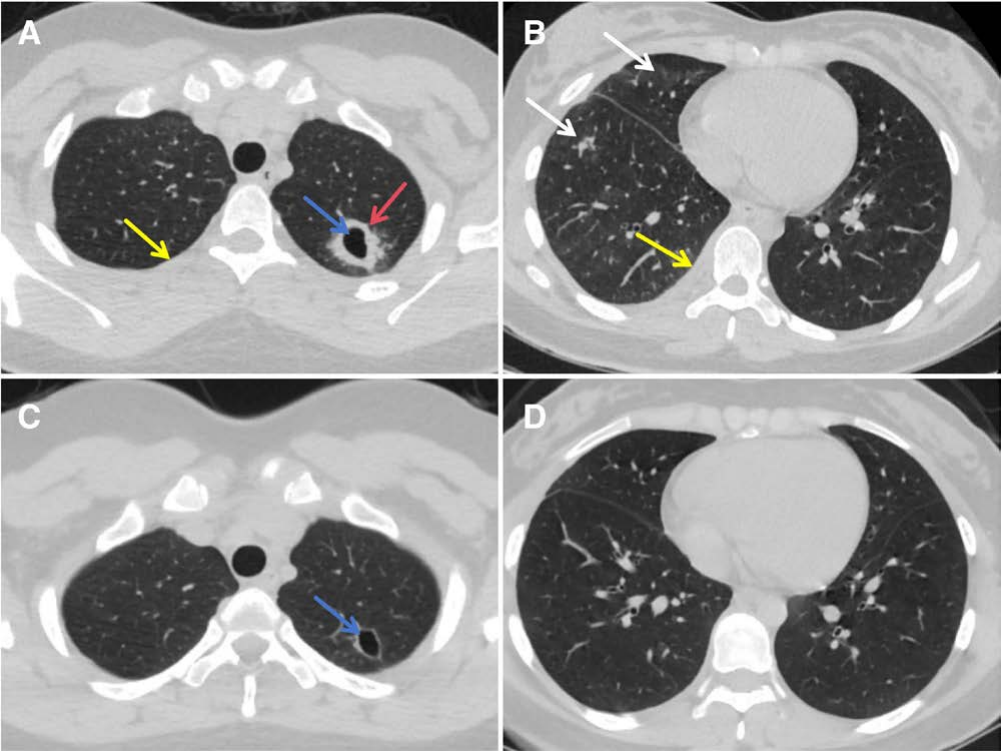
Changes in chest CT findings of the patient after a definite diagnosis of Lemierre syndrome. (A and B) After treated with meropenem, ornidazole, and closed chest drainage,the chest CT findings on June 3, 2024 indicated an enlarged cavity in the left upper lung (A, blue arrow),partial resorption of the surrounding lesion (A, red arrow), obvious absorption of pleural effusion in the right upper lung (A, yellow arrow), obvious absorption of solid lesions in the right lower lung (B, white arrows), and obvious absorption of pleural effusion in the right lower lung (B, yellow arrows); (C and D) the chest CT findings on July 15, 2024 showed that the cavity in the the left upper lung was reduced compared with the previous one (C, blue arrow), the surrounding lesion was completely absorbed, and the solid lesion and pleural effusion in the right lower lung were completely absorbed (D). CT = computed tomography.

On May 26, 2024, the empirical antibiotic therapy was initiated by the combined administration of 0.5 g azithromycin once daily and 0.1 g doxycycline twice daily. However, the patient continued to experience recurrent fever. Due to poor clinical response, the treatment regimen was changed to anti-infection treatment with administration of 0.1 g omadacycline mesylate once daily on May 27, 2024. Following the tNGS results on May 28, 2024, which identified FN and other microbessuch as *H. influenzae*, *C albicans*, *A fumigatus*, *S anginosus* (Table 1), thus the anti-infective regimen was adjusted to target the anaerobic bacterium FN with combined administration of 1 g meropenem every 8 hours and 0.5 g ornidazole twice daily, A loading dose of 400 mg voriconazole was added every 12 hours for targeted antifungal therapy against *A fumigatus*. However, the patient developed a rash following initial administration, thus 0.1 g micafungin was administered once daily for antifungal treatment against *A fumigatus*. To determine the nature of right pleural effusion indicated by the chest CT scan on May 29, 2024, therapeutic thoracentesis and drainage were performed. A pale yellow and clear pleural effusion was drained out, accompanied by a small amount of purulent fluid; the pleural effusion was sent for routine examination, biochemistry and culture, and the thoracic cavity was washed out with physiological saline. Since May 28, 2024, the patient’s symptoms, including pharyngolaryngeal pain, expectoration, hemoptysis, thoracic pain, and back pain, gradually improved, and the peak of body temperature decreased day by day. Since June 1, 2024, the patient had no fever again, the moist rales in her lungs disappeared, and the reexamination showed that the expression levels of inflammatory indicators were gradually decreased.The follow-up chest CT scan on June 3, 2024 indicated an enlarged cavity, partial absorption of the surrounding lesion in the left upper lung, obvious absorption of effusion in the right upper lung (Fig. 2A), and obvious absorption of the pleural effusion and solid lesions in the right lower lung (Fig. 2B). The patient was discharged from the hospital on June 12, 2024, and all conventional microbiological cultures (sputum culture, pleural effusion culture, and BALF culture) remained negative during this hospitalization, except the fact that both BALF tNGS and serum MetaCAP tests returned to positive results.

After being discharged from the hospital, the patient had no fever, and no discomfort symptoms such as cough and expectoration, sore throat, hemoptysis, chest tightness, shortness of breath, and neck pain.The follow-up chest CT scan on July 15, 2024 showed a smaller cavity, complete absorption of the surrounding lesion in the left upper lung (Fig. 2C) compared with the previous one, and complete absorption of effusion and solid lesion in the right lower lung (Fig. 2D). Bilateral cervical vascular ultrasound showed no abnormality.

## 3. Discussion

The patient in this case aged 17 years and had a history of chronic tonsillitis. The initial symptoms of the disease were fever, sore throat, cough, and hemoptysis, which were subsequently followed by development of pulmonary nodules, cavities, and pleural effusion. FN was rapidly identified using tNGS and MetaCAP, thereby the patient was diagnosed with LS and promptly treated with antibiotics and thoracic drainage, she ultimately achieved a full recovery.This case highlights the importance of maintaining a high index of suspicion for LS in adolescents with recurrent tonsillitis, even in the absence of jugular vein thrombosis. In 1936, Dr André Lemierre, a professor of bacteriology in Paris, published a paper on 20 cases of patients with septicemia caused by anaerobic bacteria.^[4]^ This paper laid a foundation for determining the typical clinical features of the disease that came to be known as LS. This is a rare disease with a global annual incidence of 1 per million people.^[5]^ LS occurs most often in adolescents, with an average age of onset of about 21 years^[2]^ and a male-to-female ratio of 2:1.^[6]^ Possibly due to increased resistance to antibiotics or decreased antibiotic prescriptions for upper respiratory tract infections, the incidence rate of LS has continued to increase over the past few years.^[7]^

Patients with LS have a higher risk of thromboembolic complications and death, thus timely identification and standard antibiotic treatment regimens are crucial for improving the prognosis of patients with LS. Although the core clinical features of LS are well recognized, there is currently no consensus on the definition of LS. LS is usually defined according to the following diagnostic criteria:^[1]^ oropharyngeal infection; thrombophlebitis or thrombosis of the internal jugular vein; septic emboli in distal sites, more commonly in the lungs; and FN isolated from blood culture. LS is usually accompanied by septic emboli in the lungs or other organs, and the complications include necrotizing pulmonary cavitary lesion, pleural effusions/abscesses, lung abscesses and septic embolism of large joints.^[8]^ To evaluate internal jugular vein thrombosis, ultrasound, contrast-enhanced CT or magnetic resonance imaging (MRI) can be performed. Of which, contrast-enhanced neck CT is considered the gold standard for diagnosing the internal jugular vein thrombosis.^[5]^ Although particular emphasis is placed on internal jugular vein involvement in the classic definition of LS, some patients without jugular vein thrombophlebitis have been documented in some case reports.^[9,10]^ Furthermore, growing evidence supports a broader conceptualization of LS, encompassing infectious thrombophlebitis at various vascular sites (e.g., splenic vein, azygos vein, and facial veins) following FN bacteremia.^[11–13]^ These atypical manifestations emphasize the importance of comprehensive imaging assessment for LS patients with persistent oropharyngeal symptoms and systemic manifestations, even in the absence of typical internal jugular vein thrombosis. A recent review suggested that the positive culture results of FN and the presence of distant septic embolism are as useful in the diagnosis of LS as the radiological evidence of internal jugular vein thrombosis^.[14]^ The patient had sore throat with chronic tonsillitis, purulent pleural effusion and FN indicated by pathogenetic detection, which met the diagnostic criteria of LS. The patient met all criteria for LS except for thrombophlebitis in the internal or external jugular vein. The absence of characteristic neck symptoms or a negative result of initial neck ultrasound should not dissuade clinicians from considering LS. Even in the absence of imaging evidence of jugular vein thrombophlebitis, clinicians must consider LS as a disease to be deferentially diagnosed.

The most common cause of LS is FN, an obligate anaerobic Gram-negative bacillus that is part of the normal flora in the oral cavity, gastrointestinal tract and female genital tract, which is thought to contribute to pharyngitis in older adolescents and adults under 30 years of age.^[15]^ Whereas FN is relatively common in acute tonsillitis and even more common in chronic or recurrent tonsillitis.^[16]^ Clinicians should be highly suspicious of LS especially in cases where there is a previous pharyngeal infection or tonsillitis. This case of adolescent patient has a history of recurrent tonsillitis. Therefore, it is crucial to raise physicians’ awareness of the possibility of LS in patients with prolonged upper respiratory tract infections.^[17]^ However, FN is a strictly anaerobic Gram-negative bacillus, the positive rate of traditional etiological culture is low, and this method takes a long time, thereby delaying the appropriate antibiotic treatment. Therefore, the early identification of LS is challenging. Up to now, the largest-scale cohort study of LS patients has revealed that FN is identified as the sole causative microorganism in 51% of cases. It is worth noting that another 16% of the cases involve multiple microorganisms,which are a combination of Fusobacterium and other bacterial pathogens.^[18]^ Other pathogens potentially associated with LS include streptococci, *Klebsiella pneumoniae*, Bacteroides, and *Staphylococcus aureus*.^[6]^ In the present case, BALF tNGS indicated FN, *S anginosus*, *A fumigatus*, *H. influenzae*, and *C albicans*. The patient had received broad-spectrum antibiotic therapy for several days prior to BALF sampling, which may have contributed to the low sequence counts of *H influenzae* and *S anginosus*. However, this does not rule out the presence of the initial infection. The patients with LS and *H influenzae* infection have been previously reported.^[19]^ A small number of studies have also reported LS secondary to *S anginosus* infection.^[20,21]^ An interesting report suggests that the coexistence of *S anginosus* and FN may aggravate the inflammation in LS.^[22]^ Therefore, the identification of *S anginosus* by etiological test should increase clinical suspicion for LS. The patient presented with persistent fever, cough, chest pain, hemoptysis, and pulmonary cavitation, with poor response to antibiotic treatment. At this time, BALF tNGS indicated *A fumigatus* infection. A detailed medical history review revealed that the patient had been sitting in the corner of a school classroom adjacent to a trash bin. This environment was considered a potential risk factor for *A fumigatus* exposure. Consequently, the patient was suspected to have LS complicated by fungal infection, and was thus treated with antifungal therapy. A patient with LS and *A fumigatus* infection has also been previously reported.^[23]^ *C albicans* are one of the most common fungi colonizing mucous membranes in human respiratory tract, oral cavity, and digestive tract. The patient had no definite immunosuppressive factors such as neutropenia, post-organ transplantation, and HIV infection, and no history of prolonged mechanical ventilation or ICU admission. Therefore, *C albicans* detected in the BALF were not considered as pathogenic bacteria.

The tNGS is a next-generation sequencing (NGS) technology developed by combining the targeted enrichment technology with the high-throughput sequencing technology, which can be used to detect dozens to hundreds of known pathogenic microorganisms and their virulence or antibiotic resistance genes.^[24]^ tNGS, with its advantages such as the low volume of data required, high sensitivity, low cost and fast detection speed, has become an excellent alternative to metagenomics NGS technology. In this case, the negative sputum culture at admission coupled with a lack of specific manifestations was unlikely for clinicians to diagnose LS. Fortunately, tNGS provided clues to the diagnosis of LS, and thus the patient received appropriate antibiotic treatment in time.

MetaCAP is an innovative pathogen detection technology that combines the probe capture technology with the NGS technology, which is used to detect the microbiome in different types of samples without relying on clinical cultures, and has remarkable sensitivity and specificity in detecting pathogens in sterile samples such as tissues and body fluids, with features such as broad spectrum, high sensitivity, covering entire RNA virus genome, being compatible with viral homozygous mutations, covering drug resistance sites in pathogens, and high performance-price ratio.^[25]^ In this case, FN was rapidly detected from serum using MetaCAP, which further confirmed the diagnosis of LS.

Although tNGS and MetaCAP proved valuable in this case, their limitations must be acknowledged. Both techniques may yield false-negative results in patients with prior antibiotic exposure due to reduced microbial loads. Moreover, they cannot differentiate between colonization and active infection, as demonstrated by the detection of *A fumigatus* and *C albicans* in this patient. The absence of jugular vein thrombosis on ultrasound further reminds us that radiographic findings can be inconsistent in LS. Finally, the requirement for specialized equipment and higher costs may limit accessibility in some clinical settings. Therefore, these advanced methods should serve as complementary tools rather than replacements for comprehensive clinical evaluation.

The treatment of LS requires multidisciplinary team collaboration,^[26]^ which may involve multiple disciplines such as microbiology, otolaryngology, radiology, intensive care, and hematology. Antibiotics are the mainstay of the treatment for LS. Timely antibiotic treatment of LS patients can decrease the complication and mortality rates and increase the cure rate. Current recommendations are as follows: metronidazole is used in combination with β-lactams or carbapenems for the treatment of LS. Since FN is the most extensively studied pathogenic microorganism and exhibits broad sensitivity to metronidazole.^[14,27]^ Given that the patient was an adolescent who developed nausea and vomiting after taking metronidazole, the patient was treated with ornidazole, which was associated with a lower incidence of gastrointestinal adverse effects and a better tolerability. To cover anaerobic bacteria, and some pathogenic or concurrent Gram-negative bacilli and streptococci infections, broad-spectrum carbapenem antibiotics were initially administered in empirical treatment. The patient’s condition was improved after being treated with meropenem and ornidazole, and this antibiotic regimen was continued throughout the treatment period. There is no consensus on the optimal duration of antibiotic treatment, which is usually determined based on the severity and clinical manifestations of the disease. The treatment duration in the patients was approximately 3 weeks, which was similar to that (approximately 3–5 weeks) reported in a study.^[28]^ In addition to antibiotics, the patients with LS may require surgical intervention as part of the management of septic metastatic lesions. This may include procedures such as internal jugular vein ligation, thoracic drainage, abscess drainage, mastoidectomy, tonsillectomy and invasive cranial surgery.^[29,30]^ The patient underwent thoracentesis and drainage due to right pleural effusion. The hypercoagulable state associated with FN infection carries a high risk for thrombosis. A case report suggests that anticoagulant treatment may enhance the efficacy of antimicrobial treatment for LS.^[31]^ Anticoagulant treatment is typically recommended when LS patients show no improvement within the initial 72 hours despite appropriate antibiotic and/or surgical interventions.^[5]^ Anticoagulation treatment, including oral anticoagulant treatment, proves both effective and safe for LS, which lasts for 6 to 12 weeks.^[32]^ However, a meta-analysis revealed that adding anticoagulants into the treatment regimen of LS patients did not significantly improve their mortality rate.^[33]^ Decisions regarding anticoagulation have to be individualized, and the potential benefits must be carefully balanced against the risk of bleeding.^[34]^ The use of anticoagulation in the treatment of LS remains controversial. In this case, no signs of jugular vein thrombosis were identified, and the anticoagulation treatment was not performed due to early diagnosis of LS and improved condition.

## 4. Conclusions

This case highlights the importance of the early recognition of LS in young patients with chronic tonsillitis. We propose that the combined application of tNGS and MetaCAP should be considered early in critically ill patients with suspected LS to enable rapid pathogen identification, guide precise and effective treatment, reduce the mortality rate, and ultimately improve outcomes

## Acknowledgments

We would like to express our gratitude to the patient for granting permission to use their clinical data in this paper and for the publication of this research.

## Author contributions

**Conceptualization:** Qiuyan Zhu, Qiming Liu.

**Data curation:** Qiuyan Zhu.

**Formal analysis:** Qiuyan Zhu.

**Funding acquisition:** Qiming Liu.

**Investigation:** Qiuyan Zhu.

**Methodology:** Qiuyan Zhu.

**Project administration:** Qiming Liu.

**Resources:** Qiuyan Zhu.

**Software:** Qiuyan Zhu.

**Supervision:** Qiming Liu.

**Validation:** Qiming Liu.

**Visualization:** Qiuyan Zhu.

**Writing – original draft:** Qiuyan Zhu.

**Writing – review & editing:** Qiming Liu.
